# Supplementary material for: Phylogeny of the Australian Solanum dioicum group using seven nuclear genes, with consideration of Symon’s fruit and seed dispersal hypotheses
Source: PLoS One. 2019 Apr 18;14(4):e0207564. doi: 10.1371/journal.pone.0207564 (PMC6472733; doi:10.1371/journal.pone.0207564)
Supplement: S1 Fig — Values at nodes reflect bootstrap support. (PDF) [file pone.0207564.s001.pdf]

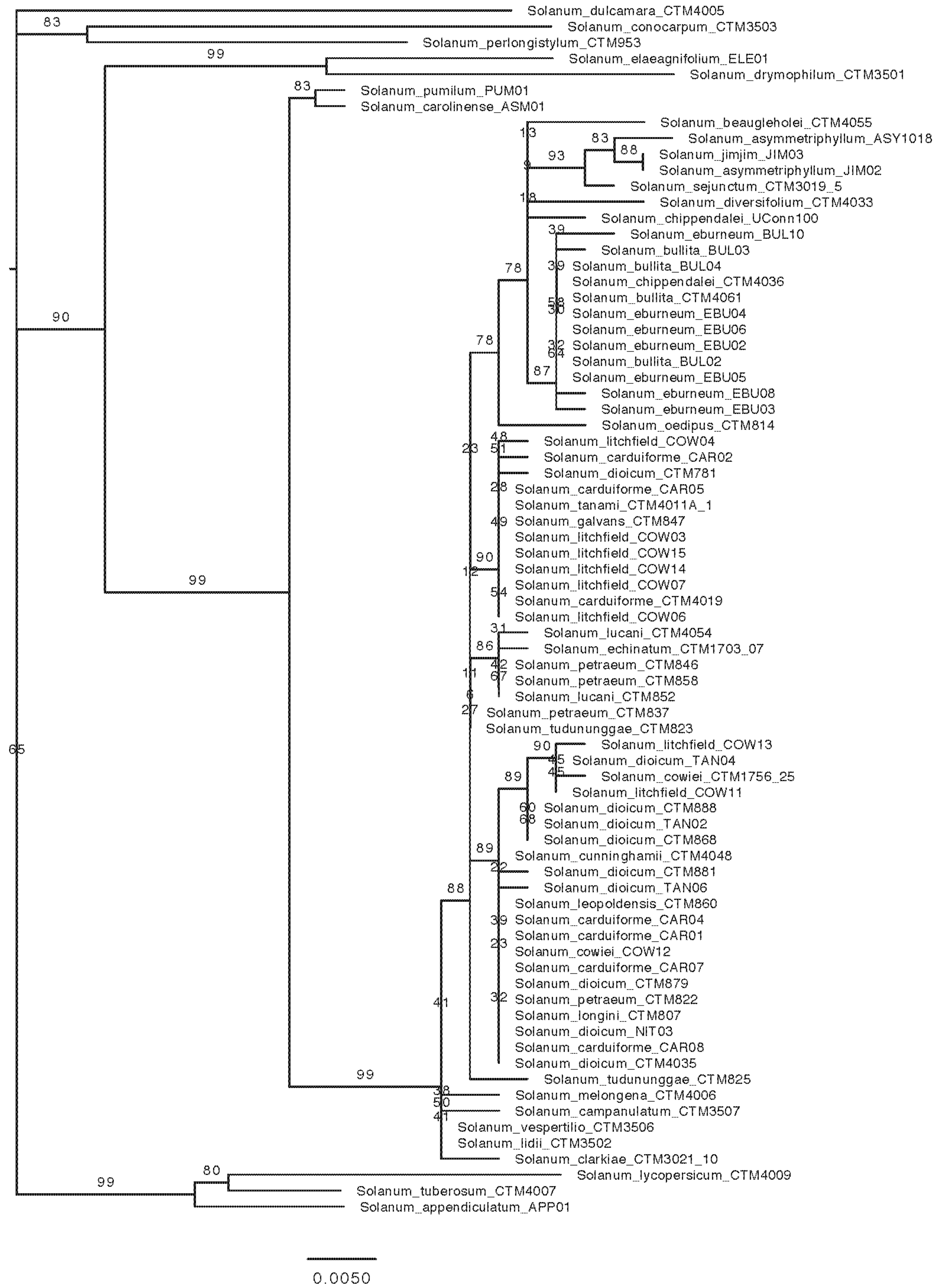
Solanum_dulcamara_CTM4005
Solanum_conocarpum_CTM3503
Solanum_perlongistylum_CTM953
Solanum_elaeagnifolium_ELE01
Solanum_drymophilum_CTM3501
Solanum_pumilum_PUM01
Solanum_carolinense_ASM01
Solanum_beaugleholei_CTM4055
Solanum_asymmetriphyllum_ASY1018
Solanum_jimjim_JIM03
Solanum_asymmetriphyllum_JIM02
Solanum_sejunctum_CTM3019_5
Solanum_diversifolium_CTM4033
Solanum_chippendalei_UConn100
Solanum_eburneum_BUL10
Solanum_bullita_BUL03
Solanum_bullita_BUL04
Solanum_chippendalei_CTM4036
Solanum_bullita_CTM4061
Solanum_eburneum_EBU04
Solanum_eburneum_EBU06
Solanum_eburneum_EBU02
Solanum_bullita_BUL02
Solanum_eburneum_EBU05
Solanum_eburneum_EBU08
Solanum_eburneum_EBU03
Solanum_oedipus_CTM814
Solanum_litchfield_COW04
Solanum_carduiforme_CAR02
Solanum_dioicum_CTM781
Solanum_carduiforme_CAR05
Solanum_tanami_CTM4011A_1
Solanum_galvans_CTM847
Solanum_litchfield_COW03
Solanum_litchfield_COW15
Solanum_litchfield_COW14
Solanum_litchfield_COW07
Solanum_carduiforme_CTM4019
Solanum_litchfield_COW06
Solanum_lucani_CTM4054
Solanum_echinatum_CTM1703_07
Solanum_petraeum_CTM846
Solanum_petraeum_CTM858
Solanum_lucani_CTM852
Solanum_petraeum_CTM837
Solanum_tudununggae_CTM823
Solanum_litchfield_COW13
Solanum_dioicum_TAN04
Solanum_cowiei_CTM1756_25
Solanum_litchfield_COW11
Solanum_dioicum_CTM888
Solanum_dioicum_TAN02
Solanum_dioicum_CTM868
Solanum_cunninghamii_CTM4048
Solanum_dioicum_CTM881
Solanum_dioicum_TAN06
Solanum_leopoldensis_CTM860
Solanum_carduiforme_CAR04
Solanum_carduiforme_CAR01
Solanum_cowiei_COW12
Solanum_carduiforme_CAR07
Solanum_dioicum_CTM879
Solanum_petraeum_CTM822
Solanum_longini_CTM807
Solanum_dioicum_NIT03
Solanum_carduiforme_CAR08
Solanum_dioicum_CTM4035
Solanum_tudununggae_CTM825
Solanum_melongena_CTM4006
Solanum_campanulatum_CTM3507
Solanum_vespertilio_CTM3506
Solanum_lidii_CTM3502
Solanum_clarkiae_CTM3021_10
Solanum_lycopersicum_CTM4009
Solanum_tuberosum_CTM4007
Solanum_appendiculatum_APP01
0.0050

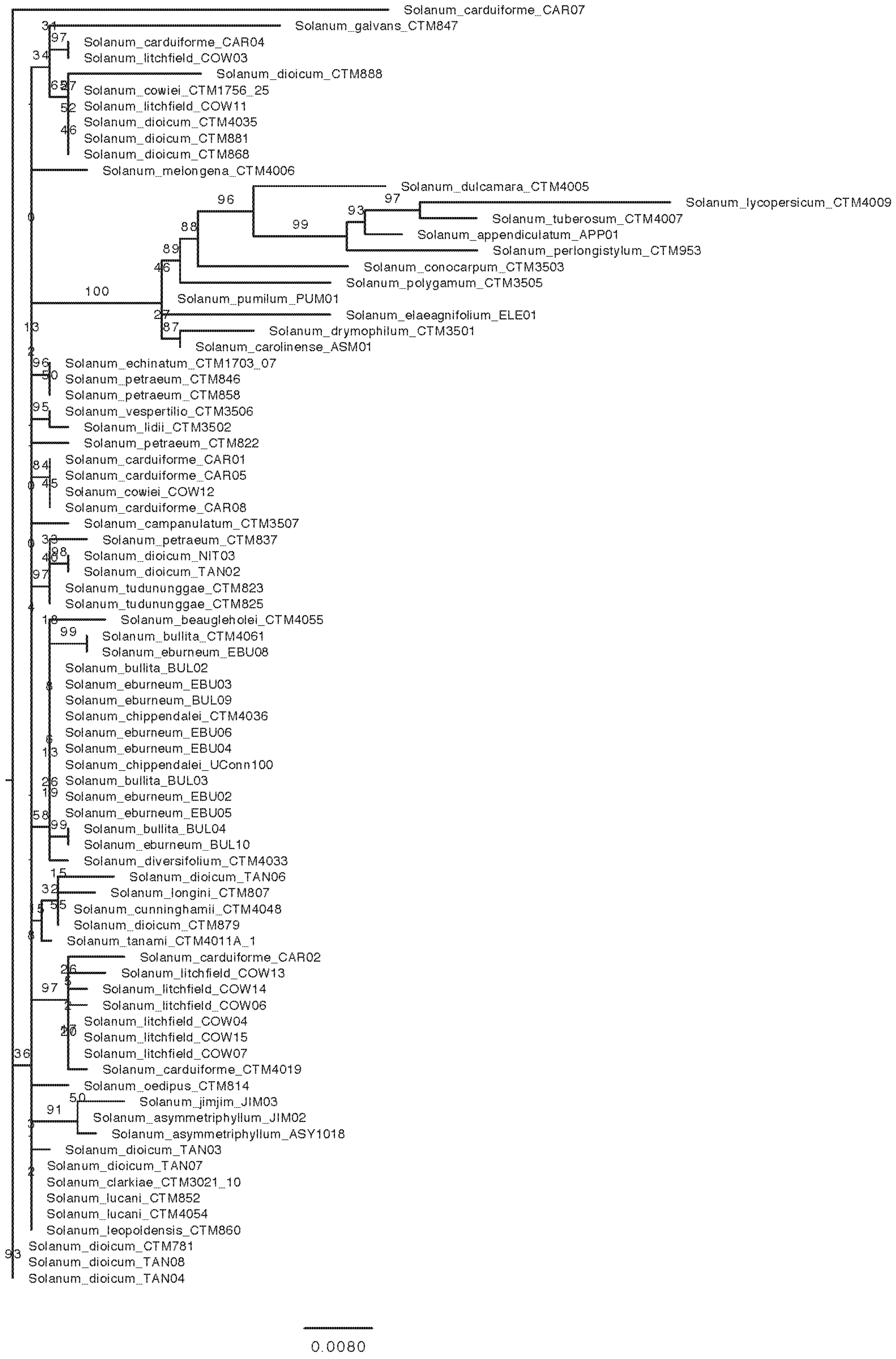

Solanum_carduiforme_CAR07
Solanum_galvans_CTM847
Solanum_carduiforme_CAR04
Solanum_litchfield_COW03
Solanum_dioicum_CTM888
Solanum_cowiei_CTM1756_25
Solanum_litchfield_COW11
Solanum_dioicum_CTM4035
Solanum_dioicum_CTM881
Solanum_dioicum_CTM868
Solanum_melongena_CTM4006
Solanum_dulcamara_CTM4005
Solanum_lycopersicum_CTM4009
Solanum_tuberosum_CTM4007
Solanum_appendiculatum_APP01
Solanum_perlongistylum_CTM953
Solanum_conocarpum_CTM3503
Solanum_polygamum_CTM3505
Solanum_pumilum_PUM01
Solanum_elaeagnifolium_ELE01
Solanum_drymophilum_CTM3501
Solanum_carolinense_ASM01
Solanum_echinatum_CTM1703_07
Solanum_petraeum_CTM846
Solanum_petraeum_CTM858
Solanum_vespertilio_CTM3506
Solanum_lidii_CTM3502
Solanum_petraeum_CTM822
Solanum_carduiforme_CAR01
Solanum_carduiforme_CAR05
Solanum_cowiei_COW12
Solanum_carduiforme_CAR08
Solanum_campanulatum_CTM3507
Solanum_petraeum_CTM837
Solanum_dioicum_NIT03
Solanum_dioicum_TAN02
Solanum_tudununggae_CTM823
Solanum_tudununggae_CTM825
Solanum_beaugleholei_CTM4055
Solanum_bullita_CTM4061
Solanum_eburneum_EBU08
Solanum_bullita_BUL02
Solanum_eburneum_EBU03
Solanum_eburneum_BUL09
Solanum_chippendalei_CTM4036
Solanum_eburneum_EBU06
Solanum_eburneum_EBU04
Solanum_chippendalei_UConn100
Solanum_bullita_BUL03
Solanum_eburneum_EBU02
Solanum_eburneum_EBU05
Solanum_bullita_BUL04
Solanum_eburneum_BUL10
Solanum_diversifolium_CTM4033
Solanum_dioicum_TAN06
Solanum_longini_CTM807
Solanum_cunninghamii_CTM4048
Solanum_dioicum_CTM879
Solanum_tanami_CTM4011A_1
Solanum_carduiforme_CAR02
Solanum_litchfield_COW13
Solanum_litchfield_COW14
Solanum_litchfield_COW06
Solanum_litchfield_COW04
Solanum_litchfield_COW15
Solanum_litchfield_COW07
Solanum_carduiforme_CTM4019
Solanum_oedipus_CTM814
Solanum_jimjim_JIM03
Solanum_asymmetriphyllum_JIM02
Solanum_asymmetriphyllum_ASY1018
Solanum_dioicum_TAN03
Solanum_dioicum_TAN07
Solanum_clarkiae_CTM3021_10
Solanum_lucani_CTM852
Solanum_lucani_CTM4054
Solanum_leopoldensis_CTM860
Solanum_dioicum_CTM781
Solanum_dioicum_TAN08
Solanum_dioicum_TAN04
0.0080

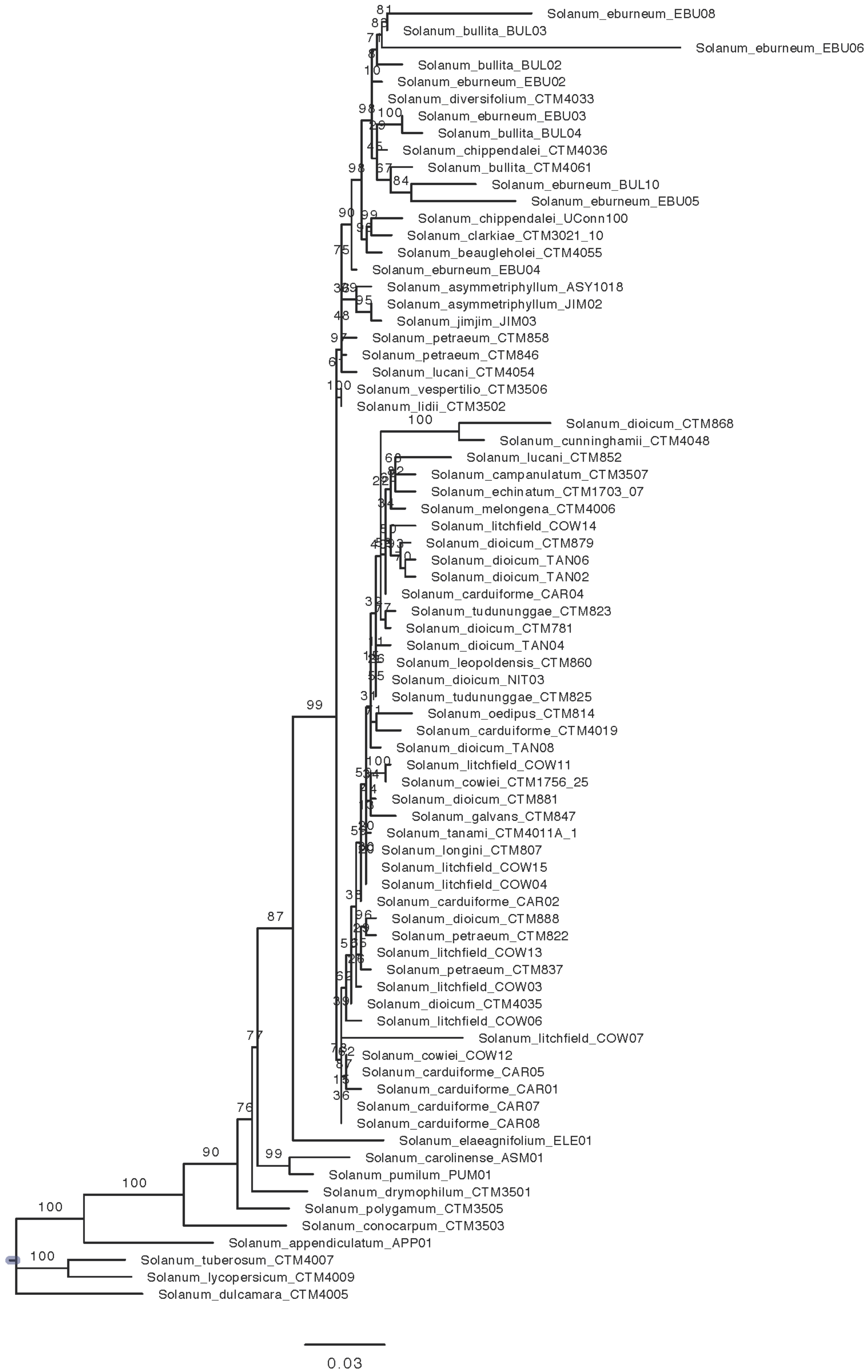
Solanum_eburneum_EBU08
Solanum_bullita_BUL03
Solanum_eburneum_EBU06
Solanum_bullita_BUL02
Solanum_eburneum_EBU02
Solanum_diversifolium_CTM4033
Solanum_eburneum_EBU03
Solanum_bullita_BUL04
Solanum_chippendalei_CTM4036
Solanum_bullita_CTM4061
Solanum_eburneum_BUL10
Solanum_eburneum_EBU05
Solanum_chippendalei_UConn100
Solanum_clarkiae_CTM3021_10
Solanum_beaugleholei_CTM4055
Solanum_eburneum_EBU04
Solanum_asymmetriphyllum_ASY1018
Solanum_asymmetriphyllum_JIM02
Solanum_jimjim_JIM03
Solanum_petraeum_CTM858
Solanum_petraeum_CTM846
Solanum_lucani_CTM4054
Solanum_vespertilio_CTM3506
Solanum_lidii_CTM3502
Solanum_dioicum_CTM868
Solanum_cunninghamii_CTM4048
Solanum_lucani_CTM852
Solanum_campanulatum_CTM3507
Solanum_echinatum_CTM1703_07
Solanum_melongena_CTM4006
Solanum_litchfield_COW14
Solanum_dioicum_CTM879
Solanum_dioicum_TAN06
Solanum_dioicum_TAN02
Solanum_carduiforme_CAR04
Solanum_tudununggae_CTM823
Solanum_dioicum_CTM781
Solanum_dioicum_TAN04
Solanum_leopoldensis_CTM860
Solanum_dioicum_NIT03
Solanum_tudununggae_CTM825
Solanum_oedipus_CTM814
Solanum_carduiforme_CTM4019
Solanum_dioicum_TAN08
Solanum_litchfield_COW11
Solanum_cowiei_CTM1756_25
Solanum_dioicum_CTM881
Solanum_galvans_CTM847
Solanum_tanami_CTM4011A_1
Solanum_longini_CTM807
Solanum_litchfield_COW15
Solanum_litchfield_COW04
Solanum_carduiforme_CAR02
Solanum_dioicum_CTM888
Solanum_petraeum_CTM822
Solanum_litchfield_COW13
Solanum_petraeum_CTM837
Solanum_litchfield_COW03
Solanum_dioicum_CTM4035
Solanum_litchfield_COW06
Solanum_litchfield_COW07
Solanum_cowiei_COW12
Solanum_carduiforme_CAR05
Solanum_carduiforme_CAR01
Solanum_carduiforme_CAR07
Solanum_carduiforme_CAR08
Solanum_elaeagnifolium_ELE01
Solanum_carolinense_ASM01
Solanum_pumilum_PUM01
Solanum_drymophilum_CTM3501
Solanum_polygamum_CTM3505
Solanum_conocarpum_CTM3503
Solanum_appendiculatum_APP01
Solanum_tuberosum_CTM4007
Solanum_lycopersicum_CTM4009
Solanum_dulcamara_CTM4005
0.03

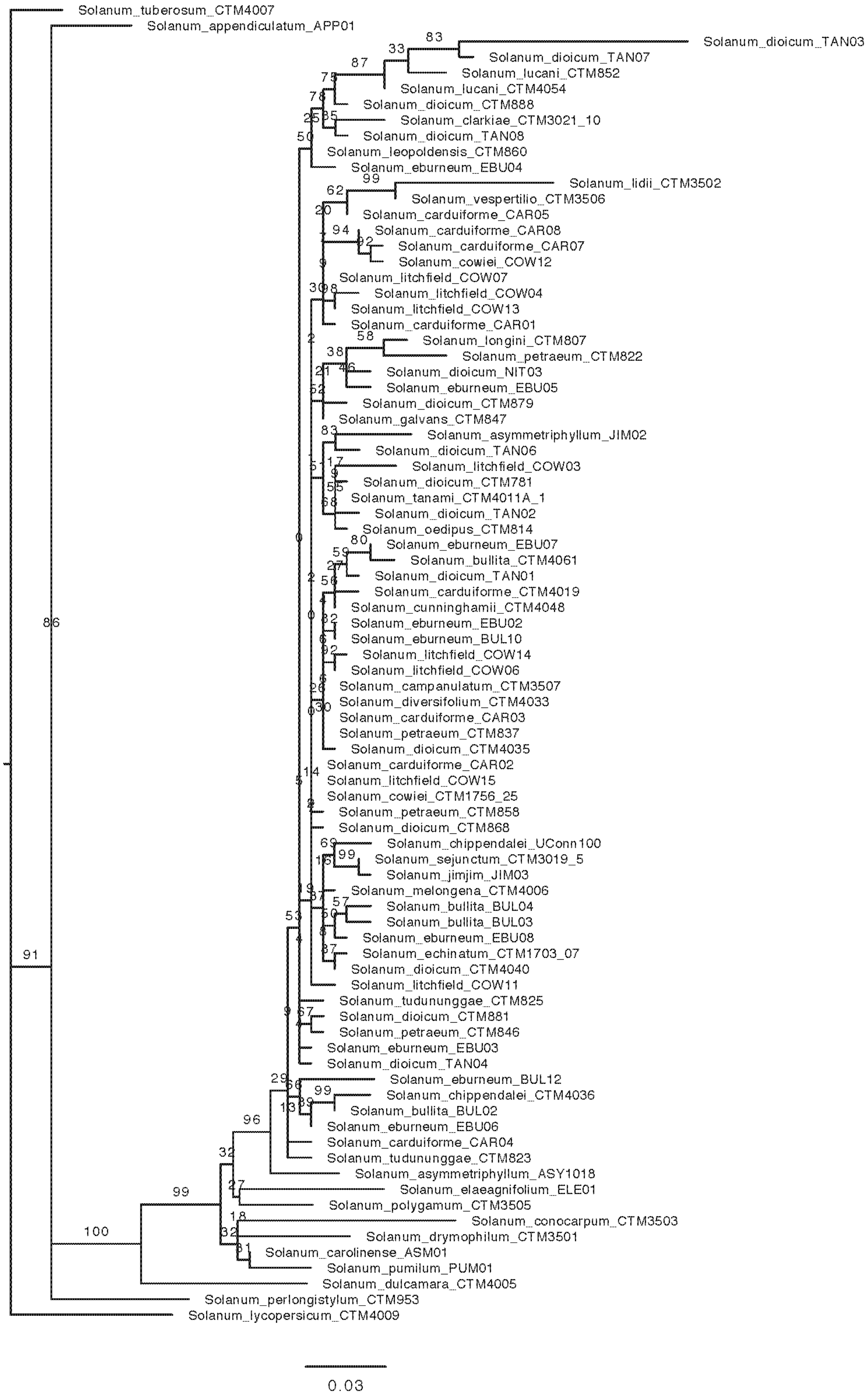
Solanum_tuberosum_CTM4007
Solanum_appendiculatum_APP01
Solanum_dioicum_TAN03
Solanum_dioicum_TAN07
Solanum_lucani_CTM852
Solanum_lucani_CTM4054
Solanum_dioicum_CTM888
Solanum_clarkiae_CTM3021_10
Solanum_dioicum_TAN08
Solanum_leopoldensis_CTM860
Solanum_eburneum_EBU04
Solanum_lidii_CTM3502
Solanum_vespertilio_CTM3506
Solanum_carduiforme_CAR05
Solanum_carduiforme_CAR08
Solanum_carduiforme_CAR07
Solanum_cowiei_COW12
Solanum_litchfield_COW07
Solanum_litchfield_COW04
Solanum_litchfield_COW13
Solanum_carduiforme_CAR01
Solanum_longini_CTM807
Solanum_petraeum_CTM822
Solanum_dioicum_NIT03
Solanum_eburneum_EBU05
Solanum_dioicum_CTM879
Solanum_galvans_CTM847
Solanum_asymmetriphyllum_JIM02
Solanum_dioicum_TAN06
Solanum_litchfield_COW03
Solanum_dioicum_CTM781
Solanum_tanami_CTM4011A_1
Solanum_dioicum_TAN02
Solanum_oedipus_CTM814
Solanum_eburneum_EBU07
Solanum_bullita_CTM4061
Solanum_dioicum_TAN01
Solanum_carduiforme_CTM4019
Solanum_cunninghamii_CTM4048
Solanum_eburneum_EBU02
Solanum_eburneum_BUL10
Solanum_litchfield_COW14
Solanum_litchfield_COW06
Solanum_campanulatum_CTM3507
Solanum_diversifolium_CTM4033
Solanum_carduiforme_CAR03
Solanum_petraeum_CTM837
Solanum_dioicum_CTM4035
Solanum_carduiforme_CAR02
Solanum_litchfield_COW15
Solanum_cowiei_CTM1756_25
Solanum_petraeum_CTM858
Solanum_dioicum_CTM868
Solanum_chippendalei_UConn100
Solanum_sejunctum_CTM3019_5
Solanum_jimjim_JIM03
Solanum_melongena_CTM4006
Solanum_bullita_BUL04
Solanum_bullita_BUL03
Solanum_eburneum_EBU08
Solanum_echinatum_CTM1703_07
Solanum_dioicum_CTM4040
Solanum_litchfield_COW11
Solanum_tudununggae_CTM825
Solanum_dioicum_CTM881
Solanum_petraeum_CTM846
Solanum_eburneum_EBU03
Solanum_dioicum_TAN04
Solanum_eburneum_BUL12
Solanum_chippendalei_CTM4036
Solanum_bullita_BUL02
Solanum_eburneum_EBU06
Solanum_carduiforme_CAR04
Solanum_tudununggae_CTM823
Solanum_asymmetriphyllum_ASY1018
Solanum_elaeagnifolium_ELE01
Solanum_polygamum_CTM3505
Solanum_conocarpum_CTM3503
Solanum_drymophilum_CTM3501
Solanum_carolinense_ASM01
Solanum_pumilum_PUM01
Solanum_dulcamara_CTM4005
Solanum_perlongistylum_CTM953
Solanum_lycopersicum_CTM4009
0.03

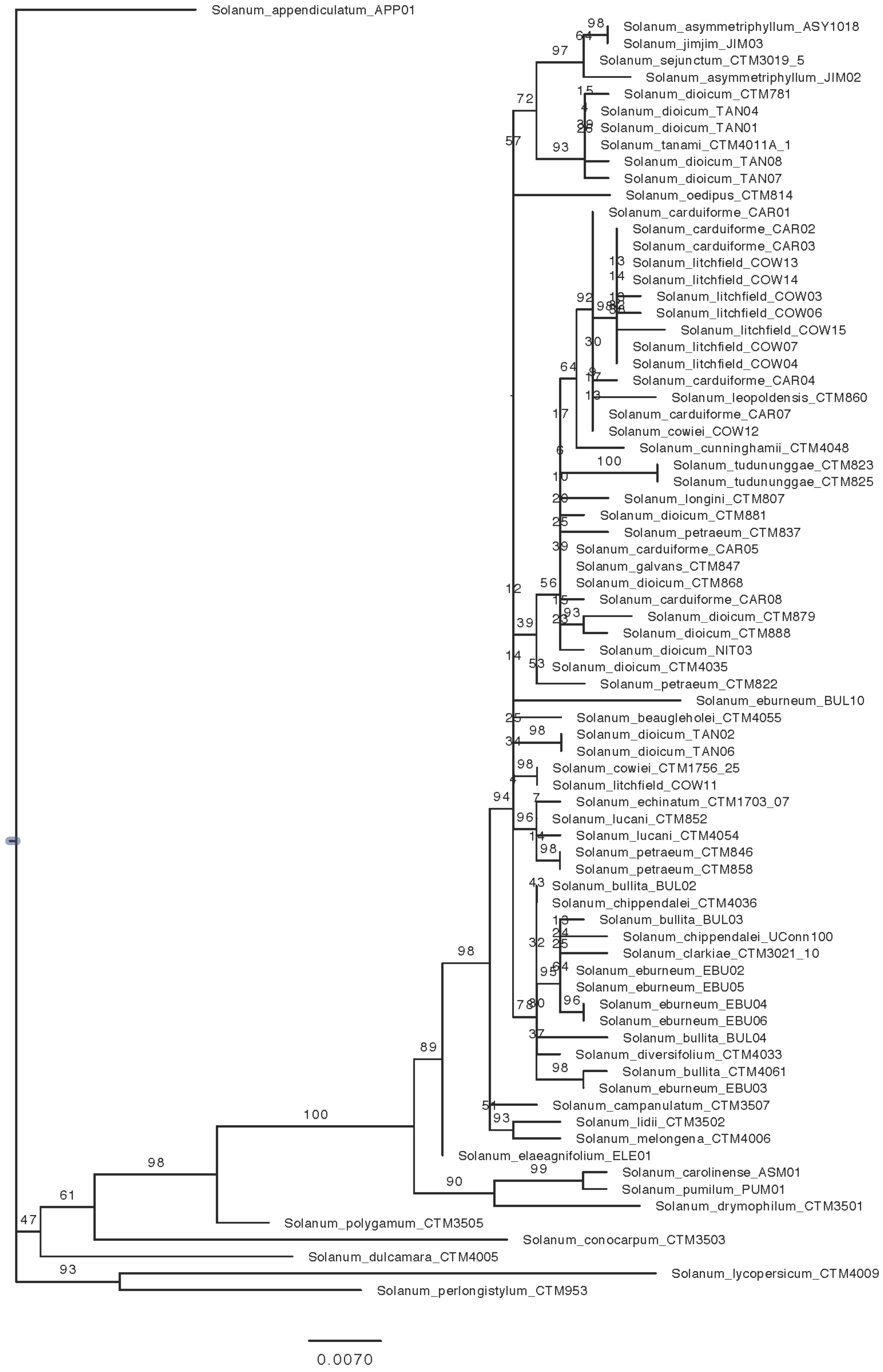
Solanum_appendiculatum_APP01
Solanum_asymmetriphyllum_ASY1018
Solanum_jimjim_JIM03
Solanum_sejunctum_CTM3019_5
Solanum_asymmetriphyllum_JIM02
Solanum_dioicum_CTM781
Solanum_dioicum_TAN04
Solanum_dioicum_TAN01
Solanum_tanami_CTM4011A_1
Solanum_dioicum_TAN08
Solanum_dioicum_TAN07
Solanum_oedipus_CTM814
Solanum_carduiforme_CAR01
Solanum_carduiforme_CAR02
Solanum_carduiforme_CAR03
Solanum_litchfield_COW13
Solanum_litchfield_COW14
Solanum_litchfield_COW03
Solanum_litchfield_COW06
Solanum_litchfield_COW15
Solanum_litchfield_COW07
Solanum_litchfield_COW04
Solanum_carduiforme_CAR04
Solanum_leopoldensis_CTM860
Solanum_carduiforme_CAR07
Solanum_cowiei_COW12
Solanum_cunninghamii_CTM4048
Solanum_tudununggae_CTM823
Solanum_tudununggae_CTM825
Solanum_longini_CTM807
Solanum_dioicum_CTM881
Solanum_petraeum_CTM837
Solanum_carduiforme_CAR05
Solanum_galvans_CTM847
Solanum_dioicum_CTM868
Solanum_carduiforme_CAR08
Solanum_dioicum_CTM879
Solanum_dioicum_CTM888
Solanum_dioicum_NIT03
Solanum_dioicum_CTM4035
Solanum_petraeum_CTM822
Solanum_eburneum_BUL10
Solanum_beaugleholei_CTM4055
Solanum_dioicum_TAN02
Solanum_dioicum_TAN06
Solanum_cowiei_CTM1756_25
Solanum_litchfield_COW11
Solanum_echinatum_CTM1703_07
Solanum_lucani_CTM852
Solanum_lucani_CTM4054
Solanum_petraeum_CTM846
Solanum_petraeum_CTM858
Solanum_bullita_BUL02
Solanum_chippendalei_CTM4036
Solanum_bullita_BUL03
Solanum_chippendalei_UConn100
Solanum_clarkiae_CTM3021_10
Solanum_eburneum_EBU02
Solanum_eburneum_EBU05
Solanum_eburneum_EBU04
Solanum_eburneum_EBU06
Solanum_bullita_BUL04
Solanum_diversifolium_CTM4033
Solanum_bullita_CTM4061
Solanum_eburneum_EBU03
Solanum_campanulatum_CTM3507
Solanum_lidii_CTM3502
Solanum_melongena_CTM4006
Solanum_elaeagnifolium_ELE01
Solanum_carolinense_ASM01
Solanum_pumilum_PUM01
Solanum_drymophilum_CTM3501
Solanum_polygamum_CTM3505
Solanum_conocarpum_CTM3503
Solanum_dulcamara_CTM4005
Solanum_lycopersicum_CTM4009
Solanum_perlongistylum_CTM953
0.0070

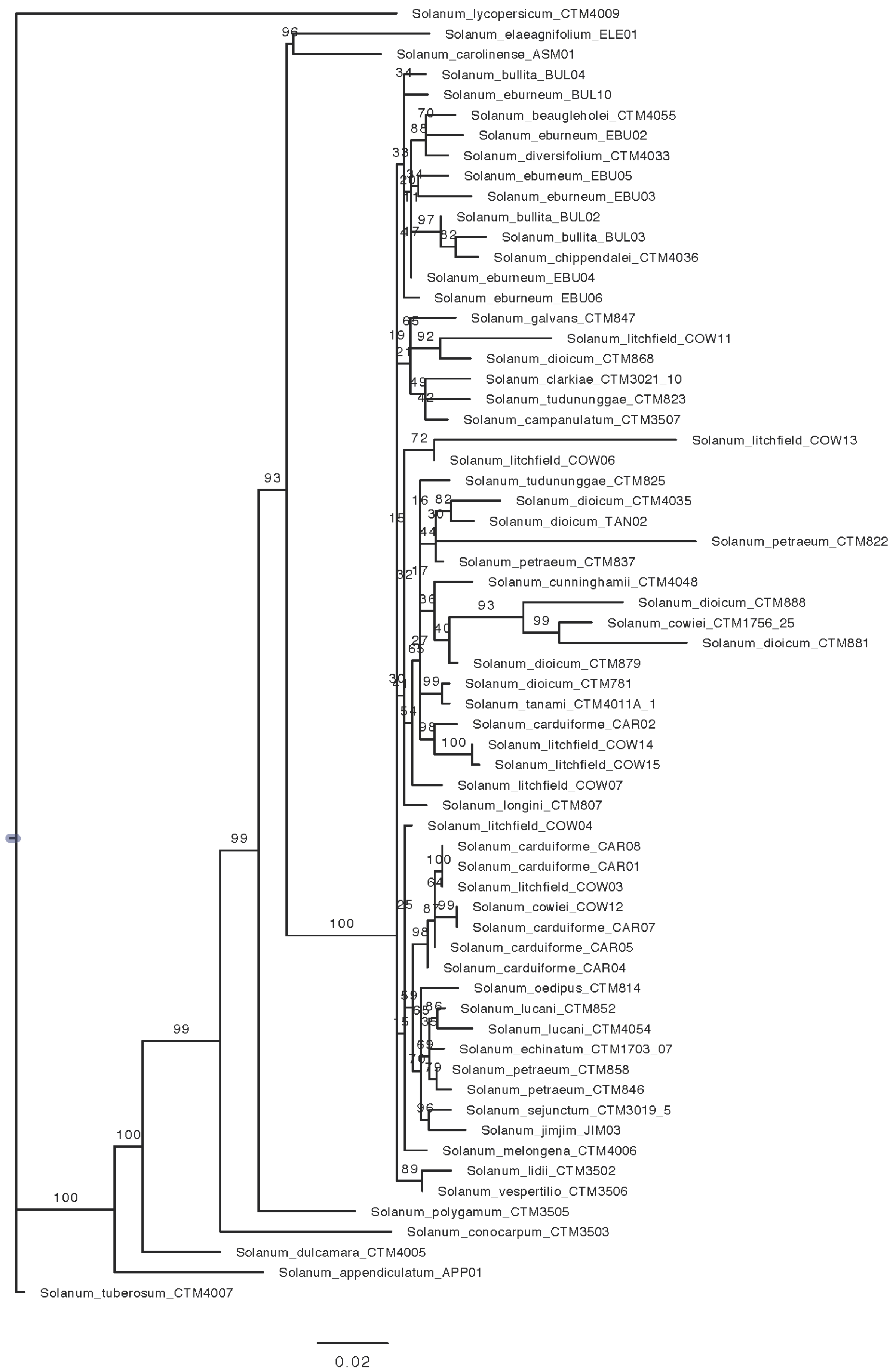

Solanum_lycopersicum_CTM4009
Solanum_elaeagnifolium_ELE01
Solanum_carolinense_ASM01
Solanum_bullita_BUL04
Solanum_eburneum_BUL10
Solanum_beaugleholei_CTM4055
Solanum_eburneum_EBU02
Solanum_diversifolium_CTM4033
Solanum_eburneum_EBU05
Solanum_eburneum_EBU03
Solanum_bullita_BUL02
Solanum_bullita_BUL03
Solanum_chippendalei_CTM4036
Solanum_eburneum_EBU04
Solanum_eburneum_EBU06
Solanum_galvans_CTM847
Solanum_litchfield_COW11
Solanum_dioicum_CTM868
Solanum_clarkiae_CTM3021_10
Solanum_tudununggae_CTM823
Solanum_campanulatum_CTM3507
Solanum_litchfield_COW13
Solanum_litchfield_COW06
Solanum_tudununggae_CTM825
Solanum_dioicum_CTM4035
Solanum_dioicum_TAN02
Solanum_petraeum_CTM822
Solanum_petraeum_CTM837
Solanum_cunninghamii_CTM4048
Solanum_dioicum_CTM888
Solanum_cowiei_CTM1756_25
Solanum_dioicum_CTM881
Solanum_dioicum_CTM879
Solanum_dioicum_CTM781
Solanum_tanami_CTM4011A_1
Solanum_carduiforme_CAR02
Solanum_litchfield_COW14
Solanum_litchfield_COW15
Solanum_litchfield_COW07
Solanum_longini_CTM807
Solanum_litchfield_COW04
Solanum_carduiforme_CAR08
Solanum_carduiforme_CAR01
Solanum_litchfield_COW03
Solanum_cowiei_COW12
Solanum_carduiforme_CAR07
Solanum_carduiforme_CAR05
Solanum_carduiforme_CAR04
Solanum_oedipus_CTM814
Solanum_lucani_CTM852
Solanum_lucani_CTM4054
Solanum_echinatum_CTM1703_07
Solanum_petraeum_CTM858
Solanum_petraeum_CTM846
Solanum_sejunctum_CTM3019_5
Solanum_jimjim_JIM03
Solanum_melongena_CTM4006
Solanum_lidii_CTM3502
Solanum_vespertilio_CTM3506
Solanum_polygamum_CTM3505
Solanum_conocarpum_CTM3503
Solanum_dulcamara_CTM4005
Solanum_appendiculatum_APP01
Solanum_tuberosum_CTM4007
0.02

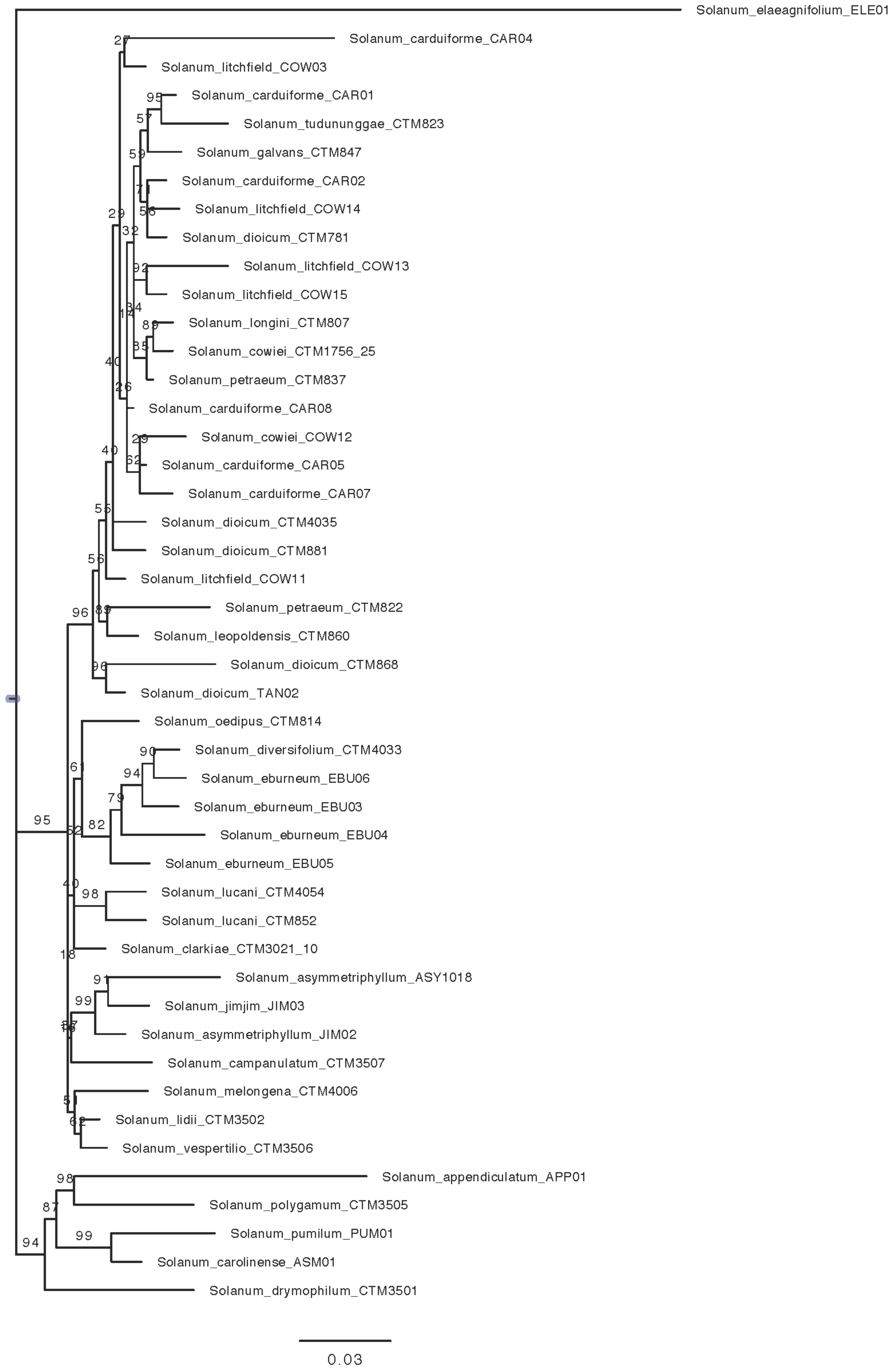
Solanum_elaeagnifolium_ELE01
Solanum_carduiforme_CAR04
Solanum_litchfield_COW03
Solanum_carduiforme_CAR01
Solanum_tudununggae_CTM823
Solanum_galvans_CTM847
Solanum_carduiforme_CAR02
Solanum_litchfield_COW14
Solanum_dioicum_CTM781
Solanum_litchfield_COW13
Solanum_litchfield_COW15
Solanum_longini_CTM807
Solanum_cowiei_CTM1756_25
Solanum_petraeum_CTM837
Solanum_carduiforme_CAR08
Solanum_cowiei_COW12
Solanum_carduiforme_CAR05
Solanum_carduiforme_CAR07
Solanum_dioicum_CTM4035
Solanum_dioicum_CTM881
Solanum_litchfield_COW11
Solanum_petraeum_CTM822
Solanum_leopoldensis_CTM860
Solanum_dioicum_CTM868
Solanum_dioicum_TAN02
Solanum_oedipus_CTM814
Solanum_diversifolium_CTM4033
Solanum_eburneum_EBU06
Solanum_eburneum_EBU03
Solanum_eburneum_EBU04
Solanum_eburneum_EBU05
Solanum_lucani_CTM4054
Solanum_lucani_CTM852
Solanum_clarkiae_CTM3021_10
Solanum_asymmetriphyllum_ASY1018
Solanum_jimjim_JIM03
Solanum_asymmetriphyllum_JIM02
Solanum_campanulatum_CTM3507
Solanum_melongena_CTM4006
Solanum_lidii_CTM3502
Solanum_vespertilio_CTM3506
Solanum_appendiculatum_APP01
Solanum_polygamum_CTM3505
Solanum_pumilum_PUM01
Solanum_carolinense_ASM01
Solanum_drymophilum_CTM3501
0.03

S1 Fig. Individual gene trees for each of the seven loci as estimated by IQ-TREE. Values at nodes reflect bootstrap support.
