# Supplementary material for: Phylogeny of the Australian Solanum dioicum group using seven nuclear genes, with consideration of Symon’s fruit and seed dispersal hypotheses
Source: PLoS One. 2019 Apr 18;14(4):e0207564. doi: 10.1371/journal.pone.0207564 (PMC6472733; doi:10.1371/journal.pone.0207564)
Supplement: S1 File — For each accession, information is as follows: collector and collector number, DNA isolate name, locality, date of collection, acronym for herbarium (in parentheses) where voucher is held, and gene regions recovered for that accession. GenBank accession numbers for gene regions used in this study are listed for each taxon in the following order, with a dash (—) inserted where a region was not recovered: SOL3, SOL8, SOL9, SOL12, SOL14, SOL15, SOL16. (PDF) [file pone.0207564.s002.pdf]

S1 File. List of specimen vouchers and GenBank numbers for sequences used in this study. For each accession, information is as follows: collector and collector number, DNA isolate name, locality, date of collection, acronym for herbarium (in parentheses) where voucher is held, and gene regions recovered for that accession. GenBank accession numbers for gene regions used in this study are listed for each taxon in the following order, with a dash (--) inserted where a region was not recovered: SOL3, SOL8, SOL9, SOL12, SOL14, SOL15, SOL16.

Solanum appendiculatum
G.J. Anderson 656, APP01, cultivated at University of Connecticut from seed collected in Mexico (41°48'37"N, 72°15'21"W), 10 August 1982 (CONN). MK071745, MK071821, MK071905, MK096182, MK096105, MK096041, MK095995.

Solanum asymmetriphyllum
C.T. Martine 3161, ASY1018, Cahill's Crosssing, Kakadu National Park, NT (12º 25.573' S, 132º 57.613' E), 15 May 2013 (BUPL). MK071749, MK071830, MK071935, MK096183, MK096106, --, MK095996.

***Solanum* aff. *asymmetriphyllum* 'Deaf Adder'**
K. Brennan 6999, JIM03, Deaf Adder Gorge, Kakadu National Park, NT (13º 06' 10" S, 132º 58' 33" E), 10 September 2006 (DNA). MK071751, MK071894, MK071937, MK096236, MK096154, MK096078, MK096022.

K. Brennan 7000, JIM02, Deaf Adder Gorge, Kakadu National Park, NT (13º 06' 10" S, 132º 58' 33" E), 10 September 2006 (DNA). MK071750, MK071831, MK071936, MK096184, MK096107, --, MK095997.

Solanum beaugleholei
C.T. Martine 4055, CTM4055, Geikie Gorge, WA (17°25'33.9"S, 124°57'49.8"E), 1 May 2014 (BUPL). MK071812, MK071888, MK071926, MK096108, --, MK096042, --.

Solanum campanulatum
C.T. Martine 3507, CTM3507, cultivated at University of Connecticut (41°48'37"N, 72°15'21"W), 2014 (BUPL). MK071766, MK071877, MK071940, MK096189, MK096113, MK096046, MK095998.

Solanum carolinense
A.S. Martinez 01, ASM01, Bucknell University campus (40°57'12"N, 76°53'07"W), 2014 (BUPL). MK071815, MK071824, MK071010, MK096198, MK096121, MK096053, MK096005.

***Solanum* aff. *carduiforme* Limmen**
B.M. Stuckey 433, CAR03-A, Billengarah Block, Limmen National Park, NT (15.93367º S, 135.58476º E), 5 August 2009 (DNA). --, --, MK096192, MK096116, --, --, --.

R.K. Harwood 684, CAR01, Nathan River Homestead, NT(15.75194º S, 135.32398º E), 19 Aug 1999 (DNA). MK071767, MK071890, MK071962, MK096190, MK096114, MK096047, MK095999.

B. Wirf 454, CAR04, St. Vigeons block, Limmen National Park, NT (15.25º S, 134.5333º E), 24 April 2009 (DNA). MK071768, MK071860, MK071963, MK096193, MK096117, MK096049, MK096001.

P.K. Latz 15041, CAR05, 25 km S. Nathan River Homestead, NT (15.799675971º S, 134.439237443º E), 24 November 1996 (DNA). MK071786, MK071891, MK071961, MK096194, MK096118, MK096050, MK096002.

K. Brennan 10019B, CAR07, Western Lost City, Limmen National Park, NT (15.76969º S, 135.35034º E), 26 September 2012 (DNA). MK071769, MK071900, MK071959, MK096195, MK096119, MK096051, MK096003.

K. Brennan 10019A, CAR08, Western Lost City, Limmen National Park, NT (15.76969º S, 135.35034º E), 26 September 2012 (DNA). MK071770, MK071892, MK071960, MK096196, MK096120, MK096052, MK096004.

***Solanum* aff. *carduiforme* Purnululu**
C.T. Martine 4019, CTM4019, Echidna Chasm, Purnululu National Park, WA (17°20’02"S, 128°24'55"E), 20 April 2014 (DNA). MK071792, MK071879, MK071969, MK071948, MK096197, --, --.

***Solanum* aff. *carduiforme* Robertson River**
K.R. McDonald 9734, CAR02, Robertson River, QL (NSW), 14 August 2010 (DNA). MK071785, MK071886, MK096191, MK096115, MK096048, MK096000.

Solanum chippendalei
C.R. Alcock 11312, Uconn100, Barkly Roadhouse, NT(19.73203º S, 136.117672º E), 17 July 1993 (CONN). MK071764, MK071835, MK071921, MK096200, MK096123, --, --.

Solanum conocarpum
C.T. Martine 3503, CTM3503, cultivated at University of Connecticut, (41°48'37"N, 72°15'21"W), 2014 (BUPL). MK071817, MK071827, MK071907, MK096202, MK096125, MK096056, --.

Solanum cowiei
C.T. Martine 1756 & D. Vogt, CTM1756-25, Litchfield National Park, NT, (13.2195º S, 130.7364º E), 27 May 2009 (CONN). MK071782, MK071872, MK071946, MK096204, MK096127, MK096058, MK096008.

K. Brennan 10019, COW12, Western Lost City, Limmen National Park, NT, (15.76969º S, 135.35034º E), 26 September 2012 (DNA). MK071771, MK071893, MK071958, --, MK096203, --, MK096126, MK096057, --, MK096007.

C.T. Martine 1754 & D. Vogt, COW11, Litchfield National Park, NT (13.2195º S, 130.7364º E), 26 May 2009 (DNA). MK071784, MK071873, MK071945, MK096243, MK096161, MK096084, MK096026.

J.O. Westaway 2368, COW14, Macadam Range, NT(14.73351º S, 129.73337º E), 16 June 2007 (DNA). MK071789, MK071883, MK071933, MK096245, MK096163, MK096086, MK096028.

I.D. Cowie 12095, COW13, Bullo River Station, NT (15.65871º S, 129.58255º E), 9 May 2008 (DNA). MK071807, MK071884, MK071955, MK096244, MK096162, MK096085, MK096027.

I.D. Cowie 11692, COW15, Spirit Hills, Keep River National Park, NT(15.31º S, 129.57º E), 18 April 2007 (DNA). MK071790, MK071882, MK071976, MK096246, MK096164, MK096087, MK096029.

I.D. Cowie 5030 & D.E. Albrecht, COW06, Fitzmaurice River Narrows, NT (14.8205º S, 129.9795º E), 14 May 1994 (DNA). MK071793, MK071885, MK071952, MK096241, MK096159, MK096082, --.

M.J.A. Barritt 1396, COW07, Fitzmaurice River, NT (14.7897º S, 130.0190º E), 14 May 1994 (DNA). MK071794,MK071881, MK071915, MK096242, MK096160, MK096083, --.

I.D. Cowie 12068, COW04, Bullo River Station, NT (15.5895º S, 129.4865º E), 7 May 2008 (DNA). MK071795,MK071880, MK071956, MK096240, MK096158, MK096081, --.

***Solanum* aff. *cowiei* Ngukurr**
R.K. Harwood 1216, COW03, 100 km N of Ngukurr, NT, (14.27º S, 134.9272º E), 28 June 2002 (DNA). MK071788, MK071861, MK071970, MK096239, MK096157, MK096080, MK096025.

Solanum cunninghamii
C.T. Martine 888 & W.R. Barker, CTM888, Bidyadanga, WA (18.7184º S, 121.8830º E), 14 June 2004 (CONN). MK071781, MK071897, MK071954, MK096212, MK096134, MK096065, --.

C.T. Martine 4048 & R.F. Martine, CTM4048, Windjana Gorge, WA (17º25'23" S, 124º55'22" E), 30 April 2014 (BUPL). MK071778, MK071857, MK071908, MK096205, MK096128, MK096059, --.

Solanum dioicum
C.T. Martine 879 & W.R. Barker, CTM879, 46 km W. of Windjana Rd., WA (17.95º S, 124.82º E), 14 June 2004 (CONN). MK071772, MK071858, MK071934, MK096210, MK096132, MK096063, --.

C.T. Martine 4040 & R.F. Martine, CTM4040, Joon Joo Botanical Trail, Derby, WA (17º20'47.7" S, 123º39'52.9" E), 25 April 2014 (BUPL). --, --, --, MK096207, --, --, --.

C.T. Martine 881 & W.R. Barker, CTM881, 22 km W. of Ellendale, WA (17.8940º S, 124.6166º E), 14 June 2004 (CONN). MK071805, MK071875, MK071967, MK096211, MK096133, MK096064, MK096012.

C.T. Martine 868 & W.R. Barker, CTM868, 20 km S. of Derby, WA (17.4343º S, 123.7441º E), 12 June 2004 (CONN). MK071779, MK071874, MK071906, MK096209, MK096131, MK096062, MK096011.

C.T. Martine 4035 & R.F. Martine, CTM4035, Ngumpan Cliff Rest Area, WA (18º44'54" S, 126º06'31" E), 22 April 2014 (BUPL). MK071773, MK071876, MK071971, MK096206, MK096129, MK096060, MK096009.

Solanum aff. dioicum Nitmiluk
B. Wirf 571, NIT03, Stuart Hwy, S. of Adelaide River, NT (13.4147º S, 131.2964º E), 31

March 2010 (DNA). MK071774, MK071864, MK071974, MK096213, MK096135, --, --.

Solanum diversiflorum
C.T. Martine 4033 & R.F. Martine, CTM4033, Wolfe Creek Crater, WA (19º10'22" S, 127º47'38" E), 21 April 2014 (BUPL). MK071811, MK071856, MK071929, MK096221, MK096142, MK096067, MK096014.

Solanum drymophilum
C.T. Martine 3501, CTM3501, cultivated at University of Connecticut from seeds supplied by Botanical Garden at Nigmegen, The Netherlands, (41°48'37"N, 72°15'21"W), 2014 (BUPL). MK071818, MK071826, MK071913, MK096222, MK096143, --, MK096015.

Solanum dulcamara
C.T. Martine 4005, CTM4005, Bucknell University campus (40°57'12"N, 76°53'07"W), 2015 (BUPL). MK071820, MK071898, MK071901, MK096223, MK096144, MK096068, --.

***Solanum* aff. *eburneum* Benmara Station**
B. Strong 253, EBU07, Benmara Station, NT (17º54' S, 136º57' E), 5 June 1984 (DNA), --, --, --, MK096231, --, --, --.

***Solanum* aff. *eburneum* Carrara Creek**
P.K. Latz 1661, BUL12, Carrara Creek, NT (18º50' S, 137º54' E), 26 July 1971 (DNA), --, --, --, MK096225, --, --, --.

***Solanum* aff. *eburneum* Jasper**
K. Brennan 9002, EBU03, Jasper Gorge, NT (16.0333º S, 130.7010º E), 27 January 2011 (DNA). MK071762, MK071837, MK071916, MK096227, MK096147, MK096071, MK096016.

***Solanum* aff. *eburneum* Savannah Way**
van der Werff & B. Gray 222501, EBU02, Savanna Way, NT (no GPS), 15 July 2008 (DNA). MK071754, MK071840, MK071930, MK096226, MK096146, MK096070, --.

***Solanum* aff. *eburneum* Wickham**
J. Russel-Smith 7739, BUL10, Wickham River, NT (16.43º S, 130.35º E), 10 March 1989 (DNA). MK071765, MK071871, MK071924, MK096224, MK096145, MK096069, --.

***Solanum* aff. *eburneum* Timber Creek**
T.R. Lally 114, EBU08, Timber Creek, NT(15º 41' S, 130º 31' E), 6 September 1993 (DNA). MK071763, MK071869, MK071904, MK096232, --, --.

Solanum echinatum
C.T. Martine 858 & W.R. Barker, CTM858, Beverley Springs Station, WA (16.7212º S, 125.4661º E), 10 June 2004 (CONN). MK071803, MK071852, MK071942, MK096257, MK096175, MK096097, --.

C.T. Martine 846 & W.R. Barker, CTM846, Barnett River Gorge, WA (16.5270º S, 126.1279º E), 8 June 2004 (CONN). MK071802, MK071851, MK071944, MK096256, MK096174, MK096096, --.

Solanum elaeagnifolium

E. Capaldi s.n., ELE01, Virgin Gorda, British Virgin Islands (no GPS), 2015 (BUPL). MK071819, MK071829, MK071909, MK096234, MK096152, MK096076, MK096020.

***Solanum* sp. Fitzroy Crossing**
C.T. Martine 4036 & R.F. Martine, CTM4036, 10 km W. of Fitzroy Crossing, WA (18º11'13" S, 125º31' 27" E), 24 April 2014 (BUPL). MK071758, MK071834, MK071925, MK096199, MK096122, MK096054, --.

***Solanum* sp. Galvans Gorge**
C.T. Martine 847 & W.R. Barker, CTM847, Galvans Gorge, WA (16.8º S, 125.8º E), June 2004 (CONN). MK071787, MK071896, MK071951, MK096235, MK096153, MK096077, MK096021.

Solanum jobsonii
B. Stuckey 595 & I.D. Cowie, EBU04, Limmen National Park, NT (16.0275º S, 135.5568º E), 8 May 2010 (DNA). MK071759, MK071838, MK071927, MK096228, MK096148, MK096072,MK096017.

I.D. Cowie 12025, EBU05, Billengarah Block, Limmen National Park, NT (15.9921º S, 135.5490º E), 23 April 2008. MK071755, MK071841, MK071931, MK096229, MK096149, MK096073,MK096018.

B. Wirf 531, EBU06, Limmen National Park, NT (15º54'56" S, 135º31'46" E), 12 May 2010 (DNA). MK071760,MK071839, MK071914, MK096230, MK096150, MK096074, --, MK096019.

Solanum leopoldense
C.T. Martine 860 & W.R. Barker, CTM860, Lennard River Gorge, WA (17º 10'44.9" S, 125º12'04.9" E), 10 June 2004 (CONN). MK071775, MK071844, MK071972, MK096237, MK096155, --, MK096023.

Solanum lidii
C.T. Martine 3502, CTM3502, cultivated at University of Connecticut from seeds collected on Canary Islands, Spain, (41°48'37"N, 72°15'21"W), 2014 (BUPL). MK071796, MK071854, MK071939, MK096238, MK096156, MK096079, MK096024.

***Solanum* sp. Longini Landing**
C.T. Martine 807 & W.R. Barker, CTM807, Longini Landing, Kalumburu, WA (14.2493º S, 126.6185º E), 1 June 2004 (CONN). MK071776, MK071859, MK071973, MK096247, MK096165, MK096088, MK096030.

Solanum lucani
C.T. Martine 4054 & R.F. Martine, CTM4054, Geikie Gorge, WA (18º05'30" S, 125º42'34" E), 1 May 2014 (BUPL). MK071804, MK071846, MK071932, MK096248, MK096166, MK096089, MK096031.

C.T. Martine 852 & W.R. Barker, CTM852, Mt. House Station, WA (16.9720º S, 125.9821º E), 9 June 2004 (CONN). MK071813, MK071845, MK071918, MK096249, MK096167, MK096090, MK096032.

Solanum lycopersicum
C.T. Martine 4009, CTM4009, Lewisburg Community Garden, Lewisburg, PA, (40°57'12"N, 76°53'07"W), 2014 (BUPL). MK071747, MK071899, MK071902, MK096250, MK096168, MK096091, --.

Solanum melongena
C.T. Martine 4006, CTM4006, Lewisburg Community Garden, Lewisburg, PA (40°57'12"N, 76°53'07"W), 2014 (BUPL). MK0717809, MK071895, MK071941, MK096251, MK096169, MK096092, MK096033.

Solanum oedipus
C.T. Martine 814 & W.R. Barker, CTM814, Kalumburu, WA (14.3º S, 126.6º E), 2 June 2004 (CONN). MK071810, MK071862, MK071919, MK096252, MK096170, MK096093, MK096034.

Solanum ossicruentum
P.K. Latz 14760, TAN08, Victoria River Range, NT (15º 40'37" S, 131º05'34" E), 16 April 1996 (DNA). --, MK071849, MK071953, MK096220, MK096141, --, --.

B. Stuckey and I.D. Cowie 64, TAN04, Bradshaw Training Area, NT (15º 04'50" S, 129º33'28" E), 16 April 1996 (DNA). MK071783, MK071848, MK071966, MK096217, MK096138, --, --.

C. Mangion 1607 & D. Lewis, TAN02, Winnecke Hills, Tanami, NT (18º 37'11" S, 130º16'30" E), 1 May 2004 (DNA). MK071780, MK071865, MK071950, MK096215, MK096137, MK096066, MK096013.

D. Edinger 2601, TAN03, Ragged Range, NT (16º 31'32"' S, 128º23'21" E), 17 July 2001 (DNA). --, MK071863, MK096216, --, --, --.

C.T. Martine 4011 & R.F. Martine, CTM4011, Mirima National Park, WA (15º 47'14.1" S, 128º45'37" E), 18 April 2014 (BUPL). MK071791, MK071853, MK071968, MK096261, MK096179, MK096100, --.

C.T. Martine 781 & W.R. Barker, CTM781, Nigli Gap, Keep River National Park, NT (15º 52'07.8" S, 129º03'11.1" E), 25 May 2004 (CONN). MK071808, MK071847, MK071965, MK096208, MK096130, MK096061, MK096010.

D. Albrecht 7756, TAN06, Jellebra Rockhole, NT (19º 21'45" S, 129º00'35" E), 7 June 1996 (DNA). MK071806, MK071889, MK071947, MK096218, MK096139, --, --.

R. Kerrigan 1226, TAN07, Spirit Hills, NT(15º 24'58" S, 129º28'39" E), 17 April 2007 (DNA). --, MK071843, --, MK096219, MK096140, --, --.

C. Mangion 1060, TAN01, Cow Creek, Gregory National Park, NT (no GPS), 2 May 2001 (DNA). --, --, --, MK096214, MK096136, --, --.

Solanum perlongistylum
C.T. Martine 953, CTM953, cultivated at University of Connecticut from seeds collected in Peru, (41°48'37"N, 72°15'21"W), 2005 (CONN). MK071748, MK071822, --, MK096253,

MK096171, --.

Solanum petraeum
C.T. Martine 822 & W.R. Barker, CTM822, Kalumburu, WA (14.1223º S, 126.7132º E), 2 June 2004 (CONN). MK071777, MK071878, MK071957, MK096254, MK096172, MK096094, MK096035.

C.T. Martine 837 & W.R. Barker, CTM837, Mertens Falls, WA (14.8241º S, 125.6983º E), 4 June 2004 (CONN). MK071798, MK071887, MK071964, MK096255, MK096173, MK096095, MK096036.

Solanum plastisexum
P.K. Latz 5482, BUL09, 92 km W. of Dunmarra, NT (16º 44' S, 132º 10' E), 20 June 1974 (DNA). --, MK071836, --, --, --, --, --.

Solanum polygamum
C.T. Martine 3505, CTM3505, cultivated at University of Connecticut from seeds collected in Puerto Rico, 2014, (41°48'37"N, 72°15'21"W), 2014 (BUPL). --, MK071828, MK071912, MK096258, MK096176, MK096098, MK096037.

Solanum pumilum
C.T. Martine s.n., PUM01, cultivated at Bucknell University from seeds collected in Alabama, US, (40°57'12"N, 76°53'07"W), 2014 (BUPL). MK071816, MK071825, MK071911, MK096259, MK096177, --, MK096038.

Solanum raphiotes
C.T. Martine 1703 & D. Vogt, CTM1703, Cahill's Crossing, Kakadu National Park, NT (12º 25.573' S, 132º 57.613' E), 16 May 2009 (DNA). MK071801, MK071850, MK071943, MK096233, MK096151, MK096075, --.

Solanum sejunctum
C.T. Martine 3019 & E. Capaldi, CTM3019, Gubara Pools Track, Kakadu National Park, NT (12º 49.787' S, 132º 52.463' E), 9 May 2013 (BUPL). MK071752, MK096260, MK096178, MK096099, --.

Solanum tuberosum
C.T. Martine 4007, CTM4007, Lewisburg Community Garden, Lewisburg, PA, (40°57'12"N, 76°53'07"W), 2014 (BUPL). MK071746, MK071823, MK071903, MK096262, --, MK096101, --.

Solanum tudununggae
C.T. Martine 823 & W.R. Barker, CTM823, Kalumburu, WA (14º18'37.8" S, 126º39'21" E), 3 June 2004 (CONN). MK071799, MK071866, MK071949, MK096263, MK096180, MK096102, MK096039.

C.T. Martine 825 & W.R. Barker, CTM825, Kalumburu, WA (14º18'37.8" S, 126º39'21" E), 3 June 2004 (CONN). MK071800, MK071867, MK071975, MK096264, MK096181, MK096103, --.

Solanum ultraspinosum
C.T. Martine 3021 & E. Capaldi, CTM3021, Gubara Pools Track, Kakadu National Park,

NT (12º 49.787’ S, 132º 52.463’ E), 9 May 2013 (BUPL). MK071814, MK071842, MK071928, MK096201, MK096124, MK096055, MK096006.

Solanum vespertilio
C.T. Martine 3506, CTM3506, cultivated at University of Connecticut from seeds collected on Canary Islands, Spain, (41°48'37"N, 72°15'21"W), 2014 (BUPL). MK071797, MK071855, MK071938, MK096265, --, MK096104, MK096040.

Solanum watneyi
M. Woodward 94 & R. Booth, BUL03, Bullock Paddock Creek, NT (16.115º S, 130.291º E), 13 April 1996 (DNA). MK071761, MK071833, MK071920, MK096186, MK096110, MK096044, --.

N. Walsh 4185, BUL04, Bullita Road, Judbarra National Park, NT (16.1158º S, 130.4239º E), 11 April 1996 (DNA). MK071756, MK071870, MK071917, MK096187, MK096111, MK096045, --.

C. Michell 491 & C. Mangion, BUL02, Limbunyah Station, NT (17.0897º S, 129.8880º E), 14 March 1997 (DNA). MK071753, MK071832, MK071922, MK096185, MK096109, MK096043, --.

C.T. Martine 4061 & R.F. Martine, CTM4061, Bullita Road, Judbarra National Park, NT (16°06.802’S, 130°25.394’E), 5 May 2014 (BUPL). MK071757, MK071868, MK071923, MK096188, MK096112, --, --.
